# Cerebral autoregulation in normal pregnancy, preeclampsia, and 1‐year postpartum

**DOI:** 10.1002/pmf2.70324

**Published:** 2026-06-01

**Authors:** Niclas Carlberg, Katja Junus, Anna‐Karin Wikström, Lina Bergman, Karusha Knipe, Catherine Cluver, Henrik Imberg, Ronney B. Panerai, Teelkien van Veen

**Affiliations:** ^1^ Department of Anesthesiology and Intensive Care Medicine Sahlgrenska University Hospital Östra Gothenburg Sweden; ^2^ Department of Obstetrics and Gynecology Institute of Clinical Sciences, Sahlgrenska Academy, University of Gothenburg Gothenburg Sweden; ^3^ Department of Women's and Children's Health Uppsala University Uppsala Sweden; ^4^ Department of Obstetrics and Gynecology Uppsala University Hospital Uppsala Sweden; ^5^ Department of Obstetrics and Gynecology Stellenbosch University Cape Town South Africa; ^6^ Department of Obstetrics and Gynecology Sahlgrenska University Hospital Gothenburg Sweden; ^7^ Statistiska Konsultgruppen Sweden AB Gothenburg Sweden; ^8^ Cerebral Hemodynamics in Ageing and Stroke Medicine Division of Cardiovascular Sciences College of Life Sciences University of Leicester Leicester UK; ^9^ NIHR Leicester Biomedical Research Centre BHF Cardiovascular Research Centre Glenfield Hospital Leicester UK; ^10^ Department of Obstetrics and Gynaecology University Medical Center Groningen the Netherlands

**Keywords:** autoregulation index, cerebral autoregulation, cerebral hemodynamics, maternal cerebrovascular physiology, preeclampsia, pregnancy complications

## Abstract

**Introduction:**

Cerebral complications of preeclampsia pose a significant threat to pregnant women worldwide. The underlying pathophysiology is unclear, but impaired cerebral autoregulation may contribute to adverse maternal outcomes. Long‐term consequences of preeclampsia include cerebrovascular disease, and impairment of cerebral autoregulation could contribute to these outcomes. The aim of this study was to assess cerebral autoregulation in women with preeclampsia and normotensive pregnancies during pregnancy and 1‐year postpartum to quantify impairment during disease and evaluate its persistence or recovery over time.

**Methods:**

This multicenter, prospective, observational cohort study included women from the UPMOST or GoPROVE studies. Women with preeclampsia were examined at diagnosis. Participants underwent transcranial Doppler examinations at inclusion and 1‐year postpartum. Women were categorized as having normotensive pregnancies or preeclampsia with or without maternal complications. Cerebral autoregulation was assessed by the autoregulation index (ARI). Differences in mean ARI between and within groups were analyzed using regression models for repeated measures.

**Results:**

A total of 124 women were included in the analysis. During pregnancy, mean ARI was higher in normotensive pregnancies compared with preeclampsia (6.90 [SD, 0.77] vs. 6.12 [SD, 1.45]; mean difference 0.78 [95% CI, 0.37–1.19]), but no difference was observed 1‐year postpartum (6.11 [SD, 1.14] vs. 6.15 [SD, 1.10]; mean difference −0.03 [95% CI, −0.47 to 0.54]). Mean ARI did not differ between preeclampsia with or without severe features, either during pregnancy (6.24 [SD, 1.49] vs. 5.98 [SD, 1.40]; mean difference 0.27 [95% CI, −0.43 to 0.96]), or 1‐year postpartum (6.13 [SD, 1.23] vs. 6.18 [SD, 0.94]; mean difference −0.05 [95% CI, −0.64 to 0.54]).

**Conclusion:**

Preeclampsia impairs the autoregulatory advantage offered by pregnancy. This renders the maternal brain more vulnerable to neurovascular injury. This impairment appears to resolve by 1‐year postpartum, suggesting that persistently depressed cerebral autoregulation is unlikely to account for long‐term cerebrovascular consequences of preeclampsia.

## 1 INTRODUCTION

Preeclampsia is a multisystem disorder of pregnancy that affects approximately 5% of pregnancies worldwide and remains a leading cause of maternal and perinatal morbidity and mortality [1]. While classically defined by new‐onset hypertension and proteinuria after 20 weeks of gestation, preeclampsia is increasingly recognized as a syndrome of endothelial dysfunction with systemic and cerebral implications. Neurological complications such as eclampsia, stroke, and posterior reversible encephalopathy syndrome are the most common lethal complications. Eclampsia (seizures in association with new‐onset hypertension in pregnancy) is still one of the most feared complications of pregnancy, with a mortality rate ranging from 1% in high‐income countries to 15% in low‐ and middle‐income countries [2].

The pathophysiology of neurological complications in preeclampsia is still unclear. Endothelial dysfunction with subsequent impaired cerebral autoregulation is likely central to the pathogenesis resulting in increased permeability across the blood–brain barrier [3, 4].

Disease‐related mechanisms leading to acute cerebrovascular events may also contribute to long‐term neurologic pathology. Long‐term risks of preeclampsia are being recognized, and within months to years after birth, there is an increased risk of developing neurological and cerebrovascular disorders [5, 6, 7, 8].

Cerebral autoregulation is a crucial function that protects the brain against blood pressure fluctuations, resulting in an approximately constant cerebral blood flow despite variations in systemic blood pressure [9]. Previous studies using transcranial Doppler ultrasound have shown an impaired cerebral autoregulation in women with preeclampsia compared to normotensive pregnant controls, with more pronounced impairments in women with severe features or neurologic symptoms, but these studies are small and might suffer from type 2 errors when comparing subtypes [10, 11]. Understanding whether impaired cerebral autoregulation resolves or persists beyond pregnancy is of both physiological and clinical importance. One study showed no differences in cerebral autoregulation between women with a history of severe preeclampsia and healthy women 1‐year postpartum [12]. However, no prospective studies have assessed the cerebral autoregulation longitudinally in women both when pregnant and nonpregnant.

We aim to assess whether depressed cerebral autoregulation in preeclampsia recovers by 1‐year postpartum. We also aim to assess whether cerebral autoregulation differs in preeclampsia with and without maternal complications compared with normotensive pregnancies.

## 2 MATERIALS AND METHODS

The study was conducted in accordance with the ethical principles of the Declaration of Helsinki. UPMOST obtained an ethical permit from the Swedish Ethical Review Authority with registration number 2019‐00309 on February 27, 2019, and GoPROVE obtained an ethical permit from the Regional Ethics Committee in Gothenburg with registration number 955–18 on December 28, 2018. Informed consent, written and oral, was obtained from all participants before entering the study.

### 2.1 Population

**FIGURE 1:**
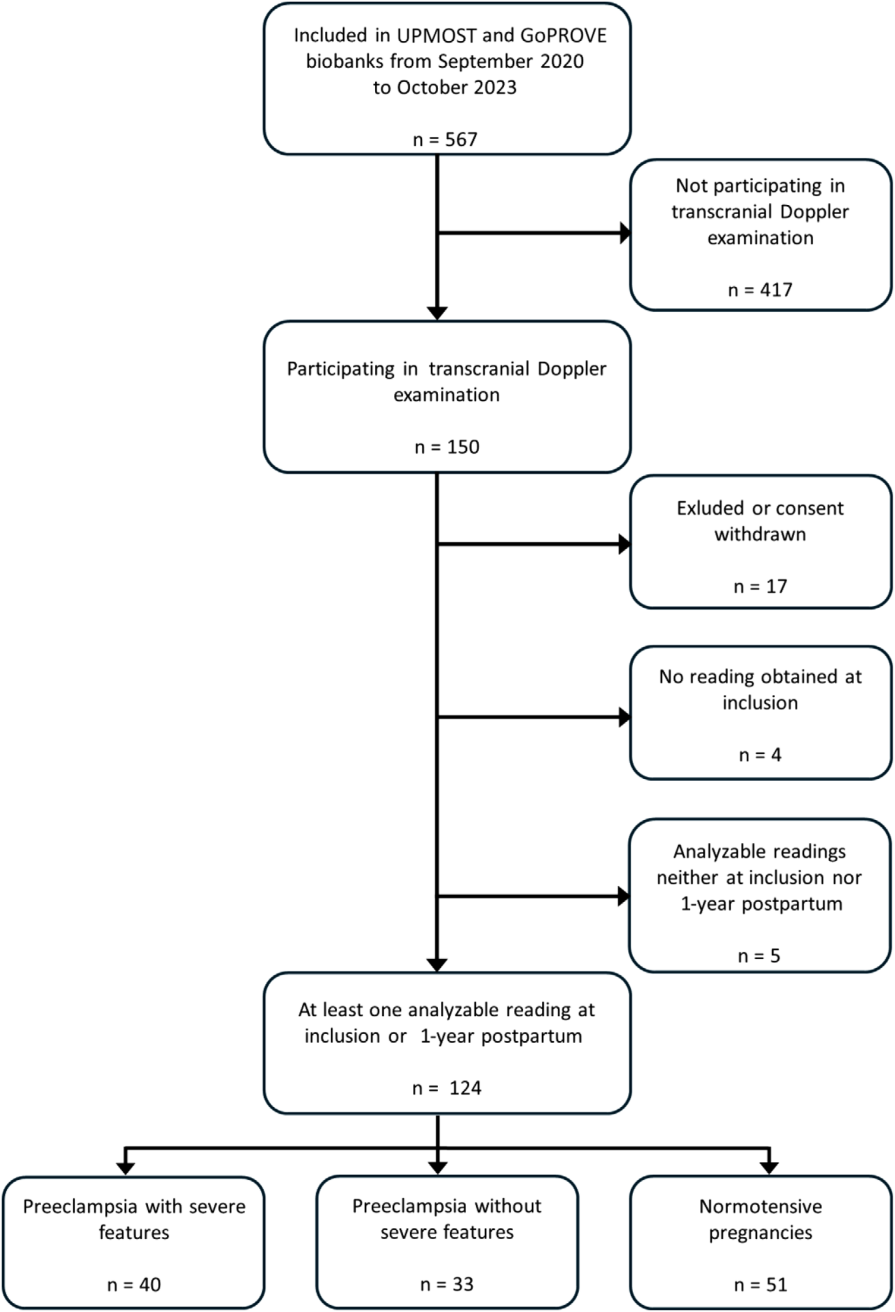
Flowchart of the study population showing the number of women undergoing transcranial Doppler examination, reasons for exclusion, and final sample analyzed.

Women with preeclampsia and normotensive pregnant women were enrolled either in the UPMOST biobank and database at Uppsala Akademiska Sjukhuset, Uppsala, Sweden or in the GoPROVE biobank and database at Sahlgrenska University Hospital Östra, Gothenburg, Sweden [13]. Both hospitals are tertiary referral centers, Uppsala Akademiska Sjukhuset with approximately 4000 deliveries per year and Sahlgrenska University Hospital Östra with approximately 10,000 deliveries per year. Women enrolled in these studies were invited to undergo transcranial Doppler examination while in hospital, provided an investigator was available and the examination could be performed without interfering with clinical care. Inclusion criteria were preeclampsia or normotensive pregnancy. Preeclampsia was defined as new‐onset hypertension (≥140/90 mmHg on two occasions at least 15 min apart) after 20 gestational weeks and excessive proteinuria, defined as a urine albumin/creatinine ratio ≥8 g/µmol, or protein/creatinine ≥30 mg/mmol, or ≥2+ protein on urine dipstick if no ratio was available. Proteinuria was required to ensure the inclusion of clearly affected cases. Normotensive pregnant women were defined as having no chronic or new‐onset hypertension, diabetes type 1 and 2, or gestational diabetes treated with medication at inclusion or later during pregnancy. Exclusion criteria were under 18 years of age, clinically significant pregestational cardiac, neurologic, or renal disease. A flow chart of the study population is presented in Figure 1. All women who participated in a primary examination agreed to be contacted again for a follow‐up examination at 1‐year postpartum. Examinations were performed at inclusion in the study, which for normotensive women could be either at any time during pregnancy or at admission for delivery, and for women with preeclampsia were performed close to hospitalization for preeclampsia. No woman was in active labor.

### 2.2 Exposure

Women were divided into groups of preeclampsia with or without maternal complications and normotensive, pregnant controls. Maternal complications were considered to be any one of several complications suggested as a core set of outcomes for preeclampsia research developed through a Delphi process, as detailed in Table S1 [14]. Information on maternal complications was retrieved from women's medical charts. All data retrieved from medical charts were primarily entered into a database by the research team and checked for accuracy by K.J. (Uppsala) and N.C. (Gothenburg).

### 2.3 Outcome

The primary outcome was autoregulation index (ARI) at follow‐up at 1‐year postpartum. Secondary outcomes were ARI at inclusion and the longitudinal change between inclusion and 1‐year postpartum follow‐up within the same woman. ARI was calculated, and measurements were performed, according to recommendations from the CARNet white paper 2022 update [15]. Examinations were performed at the maternity ward when the women were hospitalized or in research facilities at follow‐up. The same procedure was employed for all examinations, both at inclusion and 1‐year postpartum follow‐up. Women were seated in a semi‐recumbent position and instructed to relax. End‐tidal carbon dioxide (ETCO_2_) was measured with a nasal cannula connected to the Delica transcranial Doppler equipment (Shenzhen Delica Medical Equipment Co.). Blood pressure was measured continuously with a finger‐cuff and with an arm‐cuff at the start of examination using Finapres Nova (FMS medical). After calibration was achieved, AUTOCAL was turned off. Insonation of the middle cerebral artery was achieved at a depth of 45–65 mm. Flow velocity amplitudes and envelope curve waveforms confirmed the right position. Data were recorded for 7 min. Data editing and transfer function analysis (TFA) for calculation of the ARI were made using custom‐made software [15]. Data were visually inspected and periods of noise and large spikes were removed from the cerebral blood velocity (CBv) signal. All signals were filtered with a median and low‐pass filter. ETCO_2_, heart rate, mean arterial pressure, and CBvs were calculated for every cardiac cycle. TFA with the Welch method was applied to obtain coherence, phase, and gain in the low‐frequency range (less than 0.2 Hz). The inverse fast Fourier transform was performed to estimate the impulse and step responses [15]. Measurements were accepted if coherence was >0.19 [16]. We used the 10 template curves from Tiecks et al. to find the best fit for the estimated step responses [17]. These template curves correspond to ARI from 0 (absence of autoregulation) to 9 (best observed autoregulation).

### 2.4 Variables

Background data on age, parity, body mass index (registered at the first visit at the antenatal care around gestational age 12 weeks), diabetes mellitus, chronic hypertension, cardiac disease, neurological disease, and renal disease during pregnancy were retrieved from medical charts. Smoking status was retrieved from a questionnaire completed upon inclusion. Hemoglobin, platelets, creatinine, and aspartate aminotransferase were analyzed by the hospital's chemical laboratory, accredited by Swedac in accordance with ISO 15189:202, and retrieved from the medical charts. Gestational age at transcranial Doppler examination and delivery, mode of delivery, birthweight, and stillbirth were retrieved from medical charts. Information on blood pressure, blood pressure medication, magnesium therapy, and Glasgow Coma Scale was collected at the transcranial Doppler examination.

### 2.5 Statistics

Descriptive data are presented as mean (standard deviation) or median (interquartile range) for numerical variables, as appropriate, and as number (percentage) for categorical variables.

To account for the longitudinal study design and missing data, analyses of ARI and CBv were conducted using generalized least squares regression for repeated measures. An unstructured covariance matrix was specified separately by group to account for unequal variances and correlations between groups. Comparisons between groups (preeclampsia vs. normotensive, and preeclampsia with vs. without the composite outcome) as well as changes within groups (1‐year postpartum vs. during pregnancy) were performed within the same modeling framework. Results are presented as mean difference or mean changes with 95% confidence intervals (CIs). Sensitivity analyses adjusting for ETCO_2_ and magnesium therapy at the time of examination were performed by incorporating these variables as covariates in the model.

All tests were two‐tailed and conducted at the 5% significance level. All analyses were performed using SAS/STAT® Software, version 9.4 (SAS Institute Inc.).

## 3 RESULTS

Between September 2020 and October 2023, 150 out of 567 women recruited to the UPMOST and GoPROVE biobanks underwent transcranial Doppler examination. All eligible women were asked for participation during hours when the examination was feasible to perform. Eleven women were excluded as they were included as normotensive controls but later developed hypertension or proteinuria. Six women in the preeclampsia group were excluded because they did not fulfill the study diagnostic criteria for preeclampsia. In four women, no CBv‐signal in the middle cerebral artery could be obtained, and they were not called for follow‐up. At inclusion, eight readings were of insufficient quality to be analyzed, and in five of these women, no good quality readings were obtained at follow‐up; these five women were excluded from analyses (Figure 1).

Descriptive statistics of the study population are presented in Table 1. At inclusion, normotensive pregnant women were on average 31.9 (SD, 4.2) years old, whereas women with preeclampsia were 29.5 (SD, 4.2) years old. Among normotensive pregnant women, 27 out of 51 (53.0%) were nulliparous compared with 66 out of 73 (90.4%) among women with preeclampsia. Normotensive women had lower mean body mass index (24.3 [SD, 4.2] kg/m^2^ vs. 26.5 [SD, 5.8] kg/m^2^) and lower mean blood pressure (123/76 [SD, 9/6] mmHg vs. 151/94 [SD, 14/11] mmHg) compared with women with preeclampsia. At the time of transcranial Doppler examination, four women with preeclampsia (5.5%) were having magnesium infusion, and 63 out of 73 (86.3%) received antihypertensive medication, mostly oral labetalol (60 out of 73; 82.2%). There were no women with superimposed preeclampsia on chronic hypertension.

**TABLE 1:** Descriptive statistics of the study population, including women with normotensive pregnancies and women with preeclampsia, with or without the composite outcome.

Variable	Normotensive (*n* = 51)	All preeclampsia (*n* = 73)	Preeclampsia without maternal complications (*n* = 33)	Preeclampsia with maternal complications (*n* = 40)
Age (years)	31.9 (4.2)	29.5 (4.2)	29.6 (4.5)	29.3 (4.0)
Nulliparous	27 (53.0%)	66 (90.4%)	31 (93.9%)	35 (87.5%)
Multiple gestation	1 (2.0%)	0 (0%)	0 (0%)	0 (0%)
Body mass index (kg/m^2^)	24.3 (4.2)	26.5 (5.8)	26.9 (5.8)	26.1 (5.9)
Diabetes mellitus^a^	1 (2.0%)	1 (1.4%)	1 (3.0%)	0 (0%)
Chronic hypertension	0 (0%)	0 (0%)	0 (0%)	0 (0%)
Renal disease	0 (0%)	0 (0%)	0 (0%)	0 (0%)
Smoking	1 (2.0%)	3 (4.1%)	2 (6.1%)	1 (2.5%)
At inclusion				
Gestational age (weeks + days)	34+5 (30+0−37+6)	36+4 (32+1−38+1)	36+0 (30+3−38+2)	37+1 (33+0−38+2)
Systolic blood pressure (mmHg)	123 (9)	151 (14)	151 (15)	151 (14)
Diastolic blood pressure (mmHg)	76 (6)	94 (11)	93 (10)	94 (11)
Platelet count (× 10^6^)	237 (70)	188 (53)	207 (44)	173 (55)
*Missing*	*36 (70.6%)*	*0 (0%)*	*0 (0%)*	*0 (0%)*
Aspartate aminotransferase (µkat/L)	0.50 (0.03)	0.98 (1.25)	0.52 (0.23)	1.34 (1.57)
*Missing*	*49 (96.1%)*	*13 (17.8%)*	*7 (21.2%)*	*6 (15.0%)*
Creatinine (µmol/L)	50.6 (8.3)	67.4 (18.2)	60.6 (11.9)	73.6 (20.7)
*Missing*	*46 (90.2%)*	*10 (13.7%)*	*3 (9.1%)*	*7 (17.5%)*
Hemoglobin (g/dL)	11.8 (1.1)	11.2 (0.9)	11.2 (0.9)	11.2 (1.0)
*Missing*	*27 (52.9%)*	*0 (0%)*	*0 (0%)*	*0 (0%)*
Magnesium therapy	0 (0%)	4 (5.5%)	1 (3.0%)	3 (7.5%)
Blood pressure medication	0 (0%)	63 (86.3%)	28 (84.8%)	35 (87.5%)
Number of blood pressure medications	0 (0–0)	1 (1–2)	1 (1–2)	1 (1–2)
End tidal CO_2_ (mmHg)	29.7 (3.8)	28.9 (3.7)	28.8 (4.4)	28.9 (3.2)
Glasgow Coma Scale	15 (0)	15 (0)	15 (0)	15 (0)
At delivery				
Gestational age (weeks + days)	39+4 (38+6−40+3)	37+2 (34+0−38+5)	36+6 (32+2−38+3)	37+2 (34+1−38+5)
Mode of delivery				
Vaginal delivery	42 (82.4%)	33 (45.2%)	15 (45.5%)	18 (45.0%)
Cesarean section	9 (17.6%)	41 (54.8%)	18 (54.5%)	22 (55.0%)
Liveborn	51 (100%)	73 (100%)	33 (100%)	40 (100%)
Birth weight (g)	3288 (753)	2518 (1039)	2322 (1047)	2679 (1017)

At 1‐year postpartum	*n* = 31	*n* = 54	*n* = 23	*n* = 31
Time to follow‐up (years)	1.52 (0.53)	1.33 (0.46)	1.37 (0.49)	1.30 (0.44)
Systolic blood pressure (mmHg)	121 (11)	128 (10)	131 (11)	125 (9)
Diastolic blood pressure (mmHg)	74 (7)	80 (9)	82 (9)	79 (8)
End‐tidal CO_2_ (mmHg)	37.4 (6.5)	36.6 (3.4)	36.3 (4.3)	36.9 (2.8)
Blood pressure medication	0 (0%)	5 (9.3%)	3 (13.0%)	2 (6.5%)
Glasgow Coma Scale	15 (0)	15 (0)	15 (0)	15 (0)

*Note*: Maternal complications are defined as any one of the core set of outcomes for preeclampsia research defined by Duffy et al. [14]. Data are presented as mean (standard deviation) or median (interquartile range) for numeric variables and as numbers and percentages for categorical variables.

^a^ Includes diabetes mellitus type 1, type 2, and gestational diabetes.

The frequency of maternal complications is presented in Table S4. Out of 129 women called for a 1‐year follow‐up visit, 17 (32.1%) with normotensive and 15 (19.7%) with prior preeclampsia, in total 34 (26.4%), were lost to follow‐up and 95 (73.6%) attended re‐examinations on average 1.40 (SD, 0.49) years after the baseline examination. Ten readings were not analyzable. Mean systolic blood pressure was normal in both groups. None of the normotensive pregnancies were prescribed antihypertensive medication compared to 5/54 (9.3%) in women with a history of preeclampsia.

**FIGURE 2:**
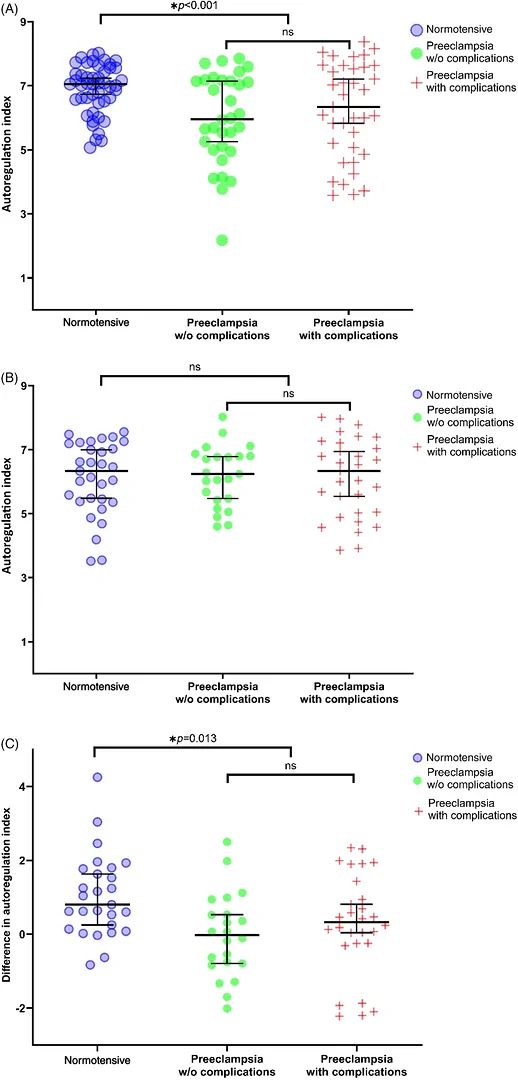
Autoregulation index (ARI) in normotensive women and women with preeclampsia, with and without maternal complications, according to the composite outcome [14]. (A) During pregnancy, (B) 1‐year postpartum, and (C) change from pregnancy to 1‐year postpartum. Black bars represent mean values, and error bars indicate 95% confidence intervals. w/o, without.

Mean ARI at inclusion was higher in normotensive pregnancies compared with preeclampsia, 6.90 (SD, 0.77) versus 6.12 (SD, 1.45), mean difference 0.78 (95% CI, 0.37–1.19), but did not differ between preeclampsia with or without maternal complications, 6.24 (SD, 1.49) versus 5.98 (SD, 1.40), mean difference 0.27 (95% CI, −0.43 to 0.96). At 1‐year postpartum, mean ARI did not differ between women with normotensive pregnancy and those with preeclampsia, 6.11 (SD, 1.14) versus 6.15 (SD, 1.10), mean difference −0.03 (95% CI, −0.47 to 0.54), nor between women with preeclampsia with or without maternal complications, 6.13 (SD, 1.23) versus 6.18 (SD, 0.94), mean difference −0.05 (95% CI, −0.64 to 0.54) (Figure 2, Tables 2 and 3).

**TABLE 2:** Cerebral autoregulation and cerebral blood velocities in normotensive pregnancies and in women with preeclampsia during pregnancy and 1‐year postpartum.

	Preeclampsia (*n* = 73)	Normotensive (*n* = 51)	Mean difference (95% CI)	*p* value
During pregnancy	*n* = 69	*n* = 47		
ARI	6.12 (1.45)	6.90 (0.77)	−0.78 (−1.19, −0.38)	<0.001
mCBv (cm/s)	65.10 (13.11)	57.20 (13.46)	7.89 (2.93, 12.86)	0.002
One‐year postpartum	*n* = 54	*n* = 31		
ARI	6.15 (1.10)	6.11 (1.14)	0.03 (−0.47, 0.54)	0.89
mCBv (cm/s)	63.37 (9.89)	63.08 (12.73)	0.29 (−4.94, 5.52)	0.91
Change from pregnancy to 1 year				
ARI	0.03 (−0.42, 0.49) *p* = 0.89^*^	−0.79 (−1.26, −0.32) *p* = 0.001^*^	0.82 (0.17, 1.47)	0.013
mCBv (cm/s)	−1.73 (−5.83, 2.38) *p* = 0.41^*^	5.87 (−0.14, 11.89) *p* = 0.055^*^	−7.60 (−14.82, −0.39)	0.039

*Note*: Descriptive data during pregnancy and 1‐year postpartum are presented as mean (SD), and changes are presented as mean differences with 95% confidence intervals (CIs). All participants were included in the analyses, including those with missing data at follow‐up. Differences between groups and changes within groups were estimated using generalized least squares regression for repeated measures, accounting for missing data under the assumption of missing at random. An unstructured covariance matrix was specified separately by group to account for unequal variances and correlations over time.

Abbreviations: ARI, autoregulation index; CBv, mean cerebral blood velocity; SD, standard deviation.

* *p* value for change from pregnancy to 1‐year postpartum.

**TABLE 3:** Cerebral autoregulation and cerebral blood velocities in women with preeclampsia with or without maternal complications during pregnancy and 1‐year postpartum.

	Preeclampsia with maternal complications (*n* = 40)	Preeclampsia without maternal complications (*n* = 33)	Mean difference (95% CI)	*p* value
During pregnancy	*n* = 37	*n* = 32		
ARI	6.24 (1.49)	5.98 (1.40)	0.27 (−0.42, 0.96)	0.45
mCBv (cm/s)	63.99 (12.49)	66.37 (13.87)	−2.38 (−8.71, 3.96)	0.46
One−year postpartum	*n* = 31	*n* = 23		
ARI	6.13 (1.23)	6.18 (0.94)	−0.05 (−0.63, 0.54)	0.87
mCBv (cm/s)	63.25 (9.82)	63.52 (10.21)	−0.27 (−5.74, 5.20)	0.92
Change from pregnancy to 1 year				
ARI	−0.11 (−0.77, 0.55) *p* = 0.73^*^	0.20 (−0.43, 0.83) *p* = 0.53^*^	−0.31 (−1.22, 0.59)	0.49
mCBv (cm/s)	−0.74 (−6.15, 4.66) *p* = 0.79^*^	−2.85 (−9.36, 3.66) *p* = 0.38^*^	2.11 (−6.26, 10.48)	0.62

*Note*: Maternal complications are defined as any one of the core set of outcomes for preeclampsia research defined by Duffy et al. [14]. Descriptive data during pregnancy and 1‐year postpartum are presented as mean (SD), and changes are presented as mean differences with 95% confidence intervals (CIs). All participants were included in the analyses, including those with missing data at follow‐up. Differences between groups and changes within groups were estimated using generalized least squares regression for repeated measures, accounting for missing data under the assumption of missing at random. An unstructured covariance matrix was specified separately by group to account for unequal variances and correlations over time.

Abbreviations: ARI, autoregulation index; CBv mean cerebral blood velocity; SD, standard deviation.

* *p* value for change from pregnancy to 1‐year postpartum.

**FIGURE 3:**
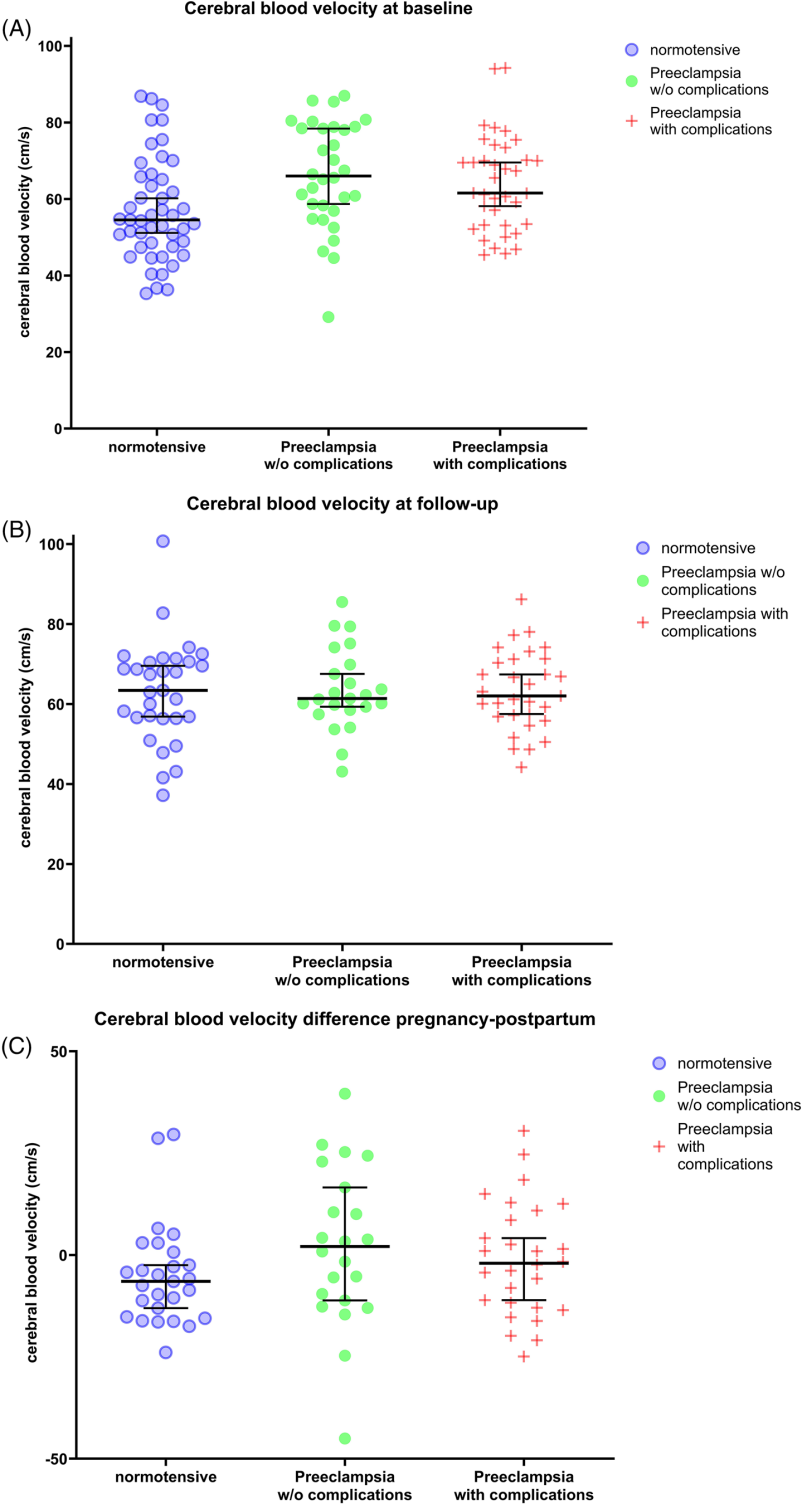
Cerebral blood velocity in normotensive women and women with preeclampsia, with and without maternal complications, according to the composite outcome [14]. (A) During pregnancy, (B) 1‐year postpartum, and (C) change from pregnancy to 1‐year postpartum. Black bars represent mean values, and error bars indicate 95% confidence intervals. w/o, without.

Differences in mean CBv followed the same pattern as differences in mean ARI and are presented in Figure 3 and Tables 2 and 3.

After adjustment for ETCO_2_ and magnesium therapy, the results remained similar (Tables S2 and S3).

## 4 DISCUSSION

At 1‐year postpartum, cerebral autoregulation was similar in women with a history of preeclampsia and in those with normotensive pregnancies. During pregnancy, cerebral autoregulation, expressed by the ARI, was lower in women with preeclampsia than in normotensive women. Longitudinally, women with preeclampsia had similar ARI during acute disease and at 1‐year postpartum, whereas normotensive women had higher ARI during pregnancy.

In our study, ARI in normotensive women was higher during pregnancy than at 1‐year postpartum, indicating enhanced cerebral autoregulation during pregnancy. This finding aligns with previous studies. In a cohort study of 76 healthy pregnant and 18 nonpregnant women, ARI was higher in pregnancy (6.7 vs. 5.3) at 20–30 weeks of gestation and remained stable through the second half of pregnancy [18]. Cerebral autoregulation can also be assessed using transfer function analysis of phase and gain. Low gain and high phase are signs of good cerebral autoregulation. In a study of 72 pregnant women at 25–28 weeks of gestation, phase was increased, and gain was unaltered compared with 26 nonpregnant controls [19].

In our study, cerebral autoregulation, expressed by the ARI, was lower in women with preeclampsia than in normotensive women. Previous work has similarly shown reduced ARI in preeclampsia (5.5 SD 1.6, *n* = 30) compared with normotensive pregnancies (6.7 SD −0.8, *n* = 30) [11]. Only one very small study suggests enhanced autoregulation in preeclampsia [20]. In summary, evidence suggests that autoregulation is depressed in preeclampsia.

Hemodynamics and cerebral blood flow are known to be altered in the early postpartum period. Miller reported increased phase and unaltered gain in all women during the first days postpartum, irrespective of preeclampsia status [21]. In another study, phase was decreased within 10 days in women with preeclampsia compared with normotensive controls, but at 6 months follow‐up, phase was similar between groups [22]. When cerebral autoregulation was assessed 2–3 years postpartum in 25 healthy women with normotensive pregnancies and 25 women with prior preeclampsia, no differences in phase or gain were observed [12]. In our study, ARI was similar at the 1‐year postpartum visit across all women, regardless of a history of normotensive pregnancy or preeclampsia with or without maternal complications. These findings suggest that in women with preeclampsia, depressed cerebral autoregulation is confined to the acute disease and normalizes by 1‐year postpartum. Therefore, long‐term cerebrovascular consequences of preeclampsia are unlikely to be explained by persistently altered cerebral autoregulation. However, our study was not designed to specifically address this question, and the data do not provide a definite basis, and it is still possible that transient impairment of cerebral autoregulation during preeclampsia could contribute to both acute and long‐term consequences.

Preeclampsia is a heterogeneous condition, and our results may not be generalizable to all subgroups. Future studies should combine assessments of cerebral autoregulation with cognitive testing, functional outcomes, and biomarkers of blood–brain barrier injury to provide further insight into both acute pathophysiologic processes and recovery after preeclampsia.

Another explanation for the comparable ARI between groups at 1‐year postpartum may be that measurements were performed in the anterior circulation, whereas most neurological symptoms of preeclampsia originate in the posterior circulation. Future studies should therefore examine both the middle and the posterior cerebral arteries.

Current approaches to ARI estimation remain complex and are not suited for timely point‐of‐care application. Development of integrated modules for point‐of‐care transfer function analysis or other modeling approaches to estimate ARI or related cerebral autoregulation indices at the time of examination would enhance the clinical utility of cerebral autoregulation in obstetrics.

### 4.1 Strengths and limitations

A strength of this study is the longitudinal design. Another strength is the multicenter setting, with data collected at two hospitals, which increases generalizability. Methodological consistency was ensured, as all examinations were done by a small number of well‐trained examiners using the same setup, reducing the risk of user‐dependent variability. However, the inability to conduct consecutive measurements on all participants recruited to UPMOST or GoPROVE introduces a potential risk of selection bias or convenience sample bias. There were fewer participants lost to follow‐up among women with preeclampsia compared with normotensive pregnancies, thereby introducing a potential risk of attrition bias. Other limitations include that most women with preeclampsia (86.3%) were already prescribed antihypertensive medication and 82.2% were prescribed labetalol at inclusion, hence we cannot distinguish the effect of labetalol from that of preeclampsia. Furthermore, information on hematocrit and caffeine consumption was lacking. A decrease in hematocrit combined with hypervolemia has been shown to increase cerebral blood flow and gain, indicating reduced cerebral autoregulation [23]. Considering that mean hemoglobin (as a proxy for hematocrit) in our study was almost the same in normotensive pregnancies and preeclampsia, this factor is unlikely to have had a substantial impact on the result. Caffeine augments cerebral autoregulation, and many studies restrict intake 4–6 h prior to assessment but this could not be accounted for in our population [24].

In our population, none of the women had eclampsia or stroke, whereby making it impossible to evaluate the association of severe neurological complications with cerebral autoregulation.

All women were contacted at 1‐year postpartum, but examinations were postponed if there was a new pregnancy or if patients canceled due to personal circumstances. There was a difference in the mean time to follow‐up between women with normotensive pregnancies and preeclampsia. It remains uncertain whether this delay may have influenced the outcome.

## 5 CONCLUSIONS

To our knowledge, this is the first study to assess cerebral autoregulation both during pregnancy and 1‐year postpartum. One‐year postpartum, cerebral autoregulation was similar between groups, while during pregnancy, cerebral autoregulation was enhanced in normotensive women but not in preeclampsia. These findings suggest that alterations in cerebral autoregulation may contribute to acute cerebral complications but persistent alterations in cerebral autoregulation are less likely to explain the long‐term cerebrovascular consequences of preeclampsia. The presence of maternal complications in preeclampsia in a high‐income setting (with no pulmonary edema, eclampsia, or HELLP in our study) does not seem to affect cerebral autoregulation.

## AUTHOR CONTRIBUTIONS

**Catherine Cluver**: Conceptualization; writing—review and editing; resources. **Katja Junus**: Conceptualization; investigation; funding acquisition; methodology; validation; writing—review and editing; data curation; supervision; resources. **Niclas Carlberg**: Conceptualization; investigation; funding acquisition; writing—original draft; methodology; validation; visualization; writing—review and editing; formal analysis; project administration; data curation. **Karusha Knipe**: Writing—review and editing; validation; conceptualization. **Anna‐Karin Wikström**: Conceptualization; funding acquisition; methodology; writing—review and editing; project administration; supervision; resources. **Lina Bergman**: Conceptualization; funding acquisition; writing—review and editing; methodology; formal analysis; project administration; supervision; resources. **Teelkien van Veen**: Conceptualization; investigation; writing—original draft; writing—review and editing; methodology; validation; formal analysis; project administration; supervision. **Henrik Imberg**: Conceptualization; formal analysis; writing—review and editing. **Ronney B. Panerai**: Conceptualization; writing—review and editing; software; supervision.

## CONFLICT OF INTEREST STATEMENT

H.I. has served as a consultant for DiaMedica Therapeutics Inc., unrelated to this work. L.B. received free reagents for sFlt‐1 and PlGF for preeclampsia projects from Thermo Fisher, Roche, and Revvity. L.B. received drugs and placebo for a randomized controlled trial in preeclampsia from Merk Damstadt, unrelated to this work. L.B. consults for DiaMedica, investigating new compounds to treat preeclampsia, unrelated to this work. C.C. has received consulting fees and research funding from DiaMedica Therapeutics Inc.; consulting fees from Beech Biotech, GondolaBio, and Avilar; and research funding from Merck KgAa Darmstadt. All funding is unrelated to this work. A.K.W. received free reagents for sFlt‐1 and PlGF for preeclampsia projects from Thermo Fisher, Roche, and Revvity. All other authors declare no conflicts of interest.

## Supporting information



Supporting Information

## Data Availability

The data that support the findings of this study are available from the corresponding author upon reasonable request.
