# Supplementary material for: Cerebral autoregulation in normal pregnancy, preeclampsia, and 1‐year postpartum
Source: Pregnancy (Hoboken). 2026 Jun 1;2(4):e70324. doi: 10.1002/pmf2.70324 (PMC13344215; doi:10.1002/pmf2.70324)
Supplement: Supplementary file 1 [file PMF2-2-e70324-s001.docx]

Supplementary material to ”Cerebral autoregulation in normal pregnancy, preeclampsia, and one-year postpartum”

**Supplementary Table S1**: The definitions of maternal complications.

| Organ dysfunction/core outcome | Definitions | |
| --- | --- | --- |
| Maternal death | | Medical chart information on maternal death |
| Eclampsia | | Generalized tonic-clonic seizures in a woman with preeclampsia (ICD code O15) |
| Stroke | | ICD codes I60, I61, I63, and I64 |
| Cortical blindness | | PRES diagnosed in medical charts, OR radiological evidence of PRES, OR diagnosis I67.83 |
| Retinal detachment | | ICD code H33.0 |
| Pulmonary edema | | Pulse oximetry ≤ 90 AND auscultatory crackles OR *x*-ray signs of pulmonary edema OR ultrasound signs of pulmonary edema |
| Acute kidney injury | | Creatinine ≥ 90 µmol/L or oliguria < 500 mL/day |
| Liver capsule hematoma or rupture | | Radiological or surgical evidence of hematoma or rupture |
| Placental abruption | | Placental abruption diagnosed in medical charts OR ICD code O45 |
| Postpartum hemorrhage | | More than 500 mL bleeding if vaginal birth OR more than 1000 mL if cesarean section |
| Raised liver enzymes | | ALT or AST ≥ 2 times highest normal value (ALT > 1.5 µkat/L and AST < 1.2 µkat/L) |
| Low platelets | | Platelet particle concentration < 100× 10^9^/L |
| Admission to ICU | | Admission to ICU |
| Intubation and mechanical ventilation | | Intubation (not for childbirth) |

Notes: ALT, alanine aminotransferase; AST, aspartate aminotransferase; ICD, international classification of diseases; ICU, intensive care unit; PRES, posterior reversible encephalopathy syndrome

## **Supplementary Table S2**: Adjusted analysis for differences in autoregulation index between women with normotensive pregnancies and in women with preeclampsia during pregnancy and one year postpartum

|  | | **Mean difference between groups (95% CI) adjusted for** | | | |
| --- | --- | --- | --- | --- | --- |
|  | | **Magnesium** | **P-value** | **ETCO2** | **P-value** |
| During pregnancy | ARI | -0.80 (-1.22, -0.37) | <.001 | -0.81 (-1.23, -0.39) | <.001 |
|  | mCBv | 8.38 (3.33, 13.43) | 0.001 | 7.85 (2.88, 12.82) | 0.002 |
| One-year postpartum | ARI | 0.03 (-0.47, 0.54) | 0.89 | 0.05 (-0.46, 0.57) | 0.83 |
|  | mCBv | 0.29 (-5.04, 5.61) | 0.91 | 0.13 (-5.24, 5.49) | 0.96 |
| Change from pregnancy to one-year postpartum | ARI | 0.83 (0.18, 1.49) | 0.013 | 0.87 (0.21, 1.53) | 0.010 |
|  | mCBv | -8.09 (-15.36, -0.82) | 0.029 | -7.72 (-14.96, -0.49) | 0.037 |
| All available observations were included in the analyses, even for individuals with single measurement. Linear mixed models were used to estimate changes over time within group and differences between group at each time point, accounting for repeated measurements within individuals. Different variances for the two groups were assumed. **Abbreviations:**  ARI, autoregulation index; mCBv mean cerebral blood velocity; CI, confidence interval. | | | | | |

**Supplementary Table S3**. Adjusted analysis for differences in autoregulation index between women with preeclampsia with or without maternal complications during pregnancy and one year postpartum

|  | | **Mean difference between groups (95% CI) adjusted for** | | | |
| --- | --- | --- | --- | --- | --- |
|  | | **Magnesium** | **P-value** | **ETCO2** | **P-value** |
| During pregnancy | ARI | 0.26 (-0.44, 0.97) | 0.46 | 0.33 (-0.39, 1.05) | 0.36 |
|  | mCBv | -1.95 (-8.37, 4.47) | 0.55 | -3.33 (-9.53, 2.88) | 0.29 |
| One-year postpartum | ARI | -0.05 (-0.64, 0.54) | 0.87 | -0.12 (-0.70, 0.46) | 0.68 |
|  | mCBv | -0.27 (-5.83, 5.29) | 0.92 | 0.34 (-5.29, 5.97) | 0.90 |
| Change from pregnancy to one-year postpartum | ARI | -0.31 (-1.22, 0.60) | 0.50 | -0.45 (-1.37, 0.46) | 0.33 |
|  | mCBv | 1.68 (-6.71, 10.08) | 0.69 | 3.67 (-4.60, 11.94) | 0.38 |
| All available observations were included in the analyses, even for individuals with single measurement. Linear mixed models were used to estimate changes over time within group and differences between group at each time point, accounting for repeated measurements within individuals. Different variances for the two groups were assumed. **Abbreviations:**  ARI, autoregulation index; mCBv mean blood velocity; CI, confidence interval. | | | | | |

## **Supplementary** **Table S4.** Frequency of maternal complications.

| **Maternal adverse event** | **n** | **% of participants** | **% of events** |
| --- | --- | --- | --- |
| All maternal adverse events | 50 | 40.3% | 100% |
| Postpartum hemorrhage | 18 | 14.5% | 36.0% |
| Elevated liver enzymes | 17 | 13.7% | 34.0% |
| Acute kidney injury | 9 | 7.3% | 18.0% |
| Low platelet count | 6 | 4.8% | 12.0% |
| Placental abruption | 0 | 0.0% | 0.0% |
| Admission to ICU | 0 | 0.0% | 0.0% |
| Eclampsia | 0 | 0.0% | 0.0% |
| Pulmonary edema | 0 | 0.0% | 0.0% |
| Cerebrovascular accident | 0 | 0.0% | 0.0% |
| Cortical blindness | 0 | 0.0% | 0.0% |
| Hepatic rupture | 0 | 0.0% | 0.0% |
| Maternal death | 0 | 0.0% | 0.0% |
| Respiratory failure | 0 | 0.0% | 0.0% |
| Retinal detachment | 0 | 0.0% | 0.0% |

**Supplementary Method Description**

Measurements were conducted on the antenatal or the labor ward or in facilities for preoperative care or in a research facility. Women were instructed to close their eyes, not talk and lean back comfortably in a semi-recumbent position. The Finapres Nova (FMS medical, Amsterdam, Netherlands) continuous non-invasive blood pressure measurement was attached to the middle finger and calibrated, while the transcranial Doppler (Shenzhen Delica Medical Equipment Co., Shenzhen, China) probes were attached with a helmet. Blood velocity readings were sought both visually and auditorily. To verify middle cerebral artery identification, we evaluated insonation depth and characteristic flow velocities. After establishing a reliable signal, automatized optimization of the signal was performed. End-tidal carbon dioxide was measured using a nasal cannula connected to the transcranial Doppler. After establishing stable signals from all measurements (transcranial Doppler blood velocities, non-invasive blood pressure and end-tidal carbon dioxide), recordings were started and continued for seven minutes. The equipment was removed and the datafiles were stored on a USB-drive.

The equipment on it’s chart, so that it can easily be brought into a delivery room on the labor ward.

The Finapres sensor is attached to the middle finger and calibrated at heart level.

All equipment attached: the helmet with the transcranial Doppler probes, the nasal cannula, the brachial blood pressure cuff, and the Finapres Nova continuous blood pressure measurement in the middle finger.

Typical transcranial Doppler assessment of blood flow velocities in the middle cerebral artery. Note the depth and the velocities (peak and mean).

A short video of a transcranial Doppler examination showing the envelope curve, velocity and depth readings, the m-mode reading, and the synchronized multimodal monitoring trace.
